# Supplementary material for: OsPSTOL but not TaPSTOL can play a role in nutrient use efficiency and works through conserved pathways in both wheat and rice
Source: Front Plant Sci. 2023 Feb 2;14:1098175. doi: 10.3389/fpls.2023.1098175 (PMC9932817; doi:10.3389/fpls.2023.1098175)
Supplement: Supplementary file 2 [file DataSheet_1.docx]

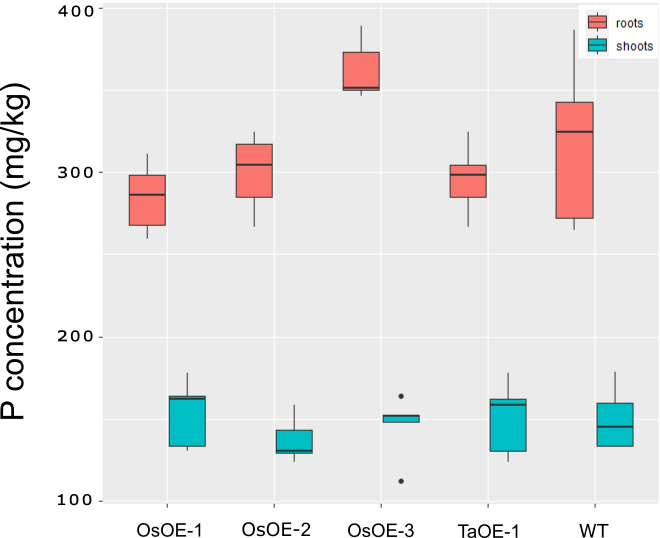


Suppl. Figure 1: Phosphorous concentration in the roots and shoots of PSTOL overexpression lines grown for two weeks in hydroponics under low P or replete nutrient solution. Data are shown as mean values (central line), lower and upper quartiles (box), minimum and maximum values (whiskers), and outliers as individual points.
